# Relationship between Serum Omega-3 Fatty Acid and Asthma Endpoints

**DOI:** 10.3390/ijerph16010043

**Published:** 2018-12-25

**Authors:** Shahieda Adams, Andreas L. Lopata, Cornelius M. Smuts, Roslynn Baatjies, Mohamed F. Jeebhay

**Affiliations:** 1 Occupational Medicine Division and Centre for Environmental and Occupational Health Research, School of Public Health and Family Medicine, University of Cape Town, Observatory, 7925 Cape Town, South Africa; shahieda.adams@uct.ac.za (S.A.); roslynn.baatjies@uct.ac.za (R.B.); 2 Molecular Allergy Research Laboratory, School of Public Health, Medical and Veterinary Sciences, James Cook University, Townsville, Douglas, QLD 4814, Australia; andreas.lopata@jcu.edu.au; 3 Centre of Excellence for Nutrition, North-West University, Potchefstroom 2520, South Africa; Marius.Smuts@nwu.ac.za; 4 Department of Environmental and Occupational Studies, Cape Peninsula University of Technology (CPUT), Cape Town 7535, South Africa

**Keywords:** non-specific bronchial hyperresponsiveness, polyunsaturated fatty acids, omega-3 fatty acids, omega-6 fatty acids, asthma clinical endpoints

## Abstract

Recent studies have highlighted the potential protective role of omega-3 polyunsaturated fatty acids (n-3 PUFA) in asthma. This study aimed at determining the association between seafood intake, serum PUFA composition and clinical endpoints of asthma in adults. A cross-sectional study of 642 subjects used the European Committee Respiratory Health Survey (ECRHS) questionnaire, skin prick tests, spirometry and methacholine challenge tests following ATS guidelines. Sera was analysed for n-3 and n-6 PUFA composition. Subjects had a mean age of 34 years, were largely female (65%) and 51% were current smokers. While 99% reported fish consumption, rock lobster, mussels, squid and abalone were also consumed less frequently. The prevalence of asthma symptoms was 11%, current asthma (ECRHS definition) was 8% and non-specific bronchial hyperresponsiveness (NSBH) was much higher (26%) In adjusted models the n-3 PUFAs 20:5 (EPA) and 22:5 (DPA) were significantly associated with a decreased risk of having NSBH. Total n-3 PUFA composition was associated with decreased NSBH risk (OR = 0.92), while high n-6 PUFA composition was associated with an increased risk (OR = 1.14).

## 1. Introduction

Asthma afflicts up to 334 million people worldwide and its incidence has been increasing for the past three decades with 250,000 deaths attributed to asthma annually [1,2]. Asthma incidence has nearly doubled in the past thirty years and despite an increase in treatment options up to 50% of asthma patients do not derive benefit from drug treatment available to them. There is therefore a growing interest in non-pharmacologic treatment options that may potentially reverse this trend. Dietary change is one of several environmental factors implicated in the rising asthma incidence and therefore represents a potentially modifiable factor to stem the tide of the asthma epidemic.

In the past two decades increasing interest has been shown in the role of dietary polyunsaturated fatty acids (PUFAs) [3,4,5]. The hypothesis underlying this assumption is linked to the dramatic change in patterns of PUFA consumption in the typical Western diet with an increasing trend for the consumption of omega-6 (n-6) PUFA mainly found in vegetable oils and a decline in consumption of omega-3 (n-3) PUFA which is mainly found in marine oils [6,7,8]. It is primarily a lower n-3PUFA dietary intake which is contributing to the increasing morbidity of asthma and allergic disease [9]. Biomarkers that better reflect this deficiency such as the red blood cell (RBC) eicosapentaenoic acid (EPA) + docosapentaenoic acid (DPA) expressed as a percentage weight of total RBC membrane fatty acids or the “omega index” have been proposed in view of the relative imprecision and lack of specificity of the ratio between n-3 and n-6 fatty acids [10].

The role of PUFA as immune modulators has been extensively studied in relation to lung health [11,12,13]. The most abundant n-6 PUFA in the Western diet, linoleic acid (LA), is converted to arachidonic acid (AA), a precursor of both prostaglandin E2 and leukotriene B4, produced by mast cells and eosinophils. Both prostaglandin and leukotriene are potent bronchoconstrictors and exhibit pro-inflammatory properties in allergic disease. In contrast alpha linolenic acid (ALA), an n-3 PUFA, is converted to EPA, which inhibits arachidonic acid and thereby suppresses production of the n-6 PUFA-derived eicosanoid inflammatory mediators. EPA functions as a precursor for bioactive molecules such as resolvins, protectins and maresins. These pro-resolving mediators have important anti-inflammatory properties [11]. Recent studies have shown that the imbalance between these pro- and anti-inflammatory molecules results in the exacerbation of inflammation as observed in the airways of asthmatics.

Research has also focused on fish intake and n-3 supplementation in relation to risk of asthma. Various studies of pregnant women have evaluated the relationship between n-3 PUFA supplementation, fish oil intake or dietary consumption and respiratory health outcomes in offspring linked to asthma. A randomized control trial demonstrated that maternal supplementation with fish oil, containing n-3 long-chain (LC)PUFA, may have prophylactic potential in the long-term prevention of asthma in offspring [13]. In a double blind RCT, Bisgaard et al. showed a reduction in the risk of persistent wheeze or asthma in offspring by approximately 7%, or one third in those offspring whose mothers received supplementation with n-3 LC-PUFA in the third trimester of pregnancy [14].

Whilst some studies have suggested that maternal fish intake during pregnancy is associated with a lowered risk of asthma among offspring in infancy and childhood, the current evidence indicates that atopic outcomes such as allergic rhinitis, wheeze and eczema are unaffected by maternal fish intake [11,15]. Fish intake in infancy has however been shown to reduce the risk of allergic rhinitis and eczema in children but not that of wheeze and asthma. The authors concluded that it is the intake of fish and not n-3-LC-PUFA which potentially has a protective effect on the risk of allergic disease in childhood and that randomized controlled trials should be conducted to confirm this [15].

Observational studies among adults, in general, have however produced inconsistent results. Several studies have shown n-3 PUFA intake to be associated with lower asthma symptom prevalence, asthma incidence or fractional exhaled nitric oxide (FeNO) levels and improved lung function [16,17,18,19]. Other studies have demonstrated that a diet high in marine n-3 PUFA (fish oil) may have beneficial effects by reducing airway hyperresponsiveness and that fish oil supplementation may reduce the severity of exercise-induced bronchoconstriction in adults [20,21]. 

In examining the relationship between fatty acids and respiratory health outcomes, studies have also investigated the frequency of dietary intake of fish or seafood as a proxy measure for n-3 PUFA consumption. A strong correlation between habitual fish intake and serum EPA and docosa-pentaenoic acid (DPA) levels has been demonstrated, suggesting that serum concentration of n-3 PUFA is a useful biomarker for dietary fish intake [22,23]. Plasma n-3 PUFA are also significantly higher in women than in men and 20% higher in fish-oil consumers compared to non-fish-oil consumers [23]. While most studies have relied on self-reporting of dietary intake to estimate n-3 PUFA intake, studies evaluating serum fatty acid composition and asthma outcomes in young adults have demonstrated consistent associations between the composition of specific n-6 fatty acids and higher asthma risk using various definitions of asthma outcomes [24]. A significant protective effect for asthma incidence related to n-3 PUFA intake has also been shown in a recent large cohort study of young adults [25].

The aim of this study was to evaluate the relationship between serum fatty acid status and various asthma endpoints in a predominantly female working population employed in fish processing factory and living in a coastal village. Various clinical endpoints of asthma were evaluated to assess the strength and consistency of associations observed.

## 2. Materials and Methods

### 2.1. Study Design, Population and Sampling

This secondary study data is nested in a cross-sectional study, which was originally designed to specifically determine the prevalence and risk factors for occupational asthma due to fish allergens in seafood processing workers. Detailed explanation of the methodology has been published elsewhere [26]. A cross-sectional study of 642 of currently employed workers was conducted in two fish processing plants working along the West Coast of South Africa. All 260 workers in Factory A were investigated. For efficiency reasons, 382 workers from Factory B were included from a total workforce of 1275 workers. These workers were chosen using stratified random sampling based on the department in which they worked. Ethical clearance of the protocol was obtained from the University of Cape Town (Ethics No. 109/99, 30/4/99), University of Michigan and the National Institute of Health (USA) prior to the study being conducted. Each participant signed informed consent prior to being tested.

### 2.2. Health Outcome Measurements

#### 2.2.1. Questionnaire

Each worker answered a standard questionnaire of the European Community Respiratory Health Survey [27]. The questionnaire was modified to include questions relating to current and previous employment, exposure to seafood aerosols (ascertained on subjective assessment of visibility of others in the work environment), tobacco smoke and patterns of seafood consumption. The questionnaire was also adapted for local conditions and translated into local languages, and back translated to assess validity and reproducibility. It was administered by trained interviewers in whichever language the worker was most fluent.

#### 2.2.2. Skin Prick Tests

Skin prick tests (SPT) were performed on each worker using standard common local aeroallergens (ALK-Abelló, A/S, Hørsholm, Denmark) that included house dust mite (*Dermatophagoides pteronyssinus*), bermuda grass (*Cynodon dactylon*), rye grass (*Lolium perenne*), cockroach (*Blatella germanica*), cat (*Felis domesticus*), dog (*Canis familiaris*), mouldmix (*Cladosporium herbarum*), *Alternaria* (*Alternata Fusarium*), *Aspergillus* (*Aspergillus fumigatus*) and mussel (*Mytilus edulis*) (LETI Alergia, Barcelona, Spain). The specific seafood extracts were prepared in-house. Histamine dihydrochloride was used as positive control and diluent of glycerol/sodium chloride as a negative control. Workers were instructed not to take any anti-histamines for three days prior to the test. Skin prick tests for atopy were administered on 625 subjects. A positive SPT was regarded as a wheal read 15 min after testing having a diameter (mean of two perpendicular measures) of 3 mm more than the negative control. Areas of wheal were traced on clear tape and stored for later verification. Atopic status was considered to be present if the SPT to one or more common aeroallergens was positive [28].

#### 2.2.3. Analysis of Serum Total Phospholipid Fatty Acid Composition

For analysis of serum total phospholipid fatty acid composition, serum was thawed and extracted with chloroform/methanol (2:1; *v*/*v*) according to a modified method of Folch et al. containing 0.01% butylated hydroxytoluene (BHT) as an antioxidant. [19] The total phospholipid band was scraped off and analysed for fatty acid composition as described previously [29]. The fatty acid methyl-esters (FAME) were identified by comparison of the retention times to those of a standard FAME mixture (Nu-Chek-Prep Inc., Elysian, MT, USA). The concentrations of individual fatty acids were measured, and the results expressed as weight percentage (% wt/wt) of the total phospholipid fatty acids (composition). The relative composition of one of the major marine n-3 PUFA, EPA (20:5n-3) was used as an index of habitual seafood consumption.

#### 2.2.4. Spirometry

American Thoracic Society (ATS) guidelines were followed for the spirometry tests [30]. Vitallograph S model bellows volume-time spirometers, calibrated at least twice a day with a three-liter syringe, were used [31]. Lung volumes obtained by spirometry were adjusted for body temperature and atmospheric pressure. Special instructions were given to workers not to smoke tobacco (at least one hour before) and to stop anti-asthmatic inhalers (4 h before) or oral asthma medications (8 h before) prior to the test. Pulmonary function reference values of the European Community for Coal and Steel (ECCS) with lower limits corresponding to the 95th percentile were used where appropriate [32]. Spirometry results with acceptable traces were obtained for 582 subjects.

#### 2.2.5. Methacholine Challenge Testing

Non-specific inhalation challenge testing using methacholine chloride powder mixed with normal saline was performed during the working day and scheduled throughout the working week using an abbreviated protocol. The two-minute tidal breathing method was used [33]. The diluent was administered using the Salter 8900 Series nebulizer set (reference 8900, Salter Labs, Arvin, CA, USA), with a nebulizer output volume 0.13 mL per minute ± 10% and particle size < 5 microns (85%).

In all subjects eligible for methacholine challenge test (MCT), saline diluent was first administered before inhalations of methacholine were done every five minutes. Subjects underwent either a short, medium or full protocol depending on the presence of asthma symptoms and baseline lung function. If the Forced Expiratory Volume in 1 s (FEV1) was 70–80% of predicted or symptoms were present, concentrations commenced at 0.03 mg/mL and doubled until 16 mg/mL (long protocol). In subjects with an asthma history or symptoms controlled and FEV1 80% of predicted, concentrations commenced at 0.125 mg/mL and doubled until 16 mg/mL (medium protocol). Those with no symptoms or history of asthma and FEV1 was 80% of predicted, concentrations of 2, 4, 8 and 16 mg/mL were used (short protocol). This short protocol procedure was completed in 35 min. A positive methacholine challenge test with a PC20 8 mg/mL was considered highly suggestive of asthma [34]. Subjects in whom MCT was contraindicated, such as those with acute asthma symptoms or a baseline FEV1 < 1.5 L or FEV1 < 70% predicted, a bronchodilator (400 µg salbutamol) was administered instead [35]. A change in FEV1 of 12% and 180 mL increase after 10 min of bronchodilator administration was considered to confirm the presence of NSBH. The methacholine challenge tests were conducted on 542 subjects.

### 2.3. Definitions

Key associations of interest involved investigating the relationships between seafood intake, serum polyunsaturated fatty acids and clinical endpoints for asthma. Definitions of the key clinical outcome variables are presented below and include the previously validated operational definition of current asthma as used in the European Community Respiratory Health study (ECHRS), which is widely used in asthma prevalence surveys [27].

#### 2.3.1. Predictor Variables

Data on frequency of fish and seafood intake were collected in a standardized format and coded in a uniform manner. Serum polyunsaturated fatty acid levels were reported as per laboratory analysis and expressed as the weight % of total phospholipid composition.

#### 2.3.2. Asthma-Related Endpoints of Interest

Atopy: A positive SPT with a wheal read 15 min after testing that had a diameter (mean of two perpendicular measures) of ≥3 mm more than the negative control to any allergen extract.

Asthma symptoms: Affirmative response to any of: “Have you had an attack of asthma in the last 12 months?”, “Have you been woken by an attack of shortness of breath in the last 12 months?”, “Have you been woken up with a feeling of tightness in your chest any time in the last 12 months?”

Current as asthma (ECRHS): Presence of either an attack of shortness of breath, an attack of asthma or use of asthma medication.

Non-specific bronchial hyperresponsiveness: A positive methacholine challenge test with a PC20 ≤ 8 mg/mL causing a drop in FEV1 of 20% or more or an increase in FEV1 of ≥12% after 10 min of bronchodilator administration on baseline spirometry.

#### 2.3.3. Covariates

Standard questionnaires were used to maintain consistency in the assessment of demographic information, including data on age, and sex. Smoking status was classified into three groups: current, former, or never.

### 2.4. Data Analysis

Statistical analyses were performed using STATA version 8 computer software (Stata Corp, College Station, TX, USA) [36]. The general approach involved univariate, bivariate and multivariate analyses of the outcomes of interest in relation to the predictors of interest. Generalized linear models were used for logistic regression analyses with individual dichotomous outcomes. Key associations of interest involved investigating the relationships between host factor attributes (age, gender, smoking, seafood intake and serum fatty acid levels), in relation to atopy, non-specific bronchial hyperresponsiveness and asthma clinical outcomes using bivariate unadjusted models. Separate multivariate logistic regression models adjusting for age, gender and smoking were used to examine the role of specific serum PUFAs in relation to asthma clinical endpoints. The serum concentrations of PUFA were treated as a continuous variable and were analysed using logistic regression since the asthma outcomes used were binary. The resulting risk estimates (odds ratio) are based on a one unit change in measured serum fatty acid.

## 3. Results

The study population comprised 642 workers from two seafood processing factories located in a small fishing village on the West Coast of South Africa. The study subjects had a mean age of 35 years. A large proportion of the workers investigated were female (64%) and lived in the village with a low socio-economic background (see Table 1).

A high prevalence of smoking was found with 327 (51%) workers being current smokers at the time of the survey. The population also reported a high prevalence of previous respiratory disease with 13% reported being treated for tuberculosis in the past. Although a considerable proportion (23%) reported ocular nasal symptoms, very few (2%) were receiving medical treatment for allergic disease such as asthma or allergic rhinitis at the time of the survey. There were 7% of participants who reported having doctor diagnosed asthma.

There were some significant differences found between males and females following stratified analysis. Females reported higher rates of treatment for bronchitis (chi 5.94; *p* = 0.015) and NSBH (chi 19.55; *p ≤* 0.001) and lower rates of smoking (chi 16.70; *p* < 0.001) and childhood hayfever (chi 23.27; *p* < 0.001).

A substantial proportion of the population were atopic (37%), with sensitization to indoor aeroallergens such as house dust mite (25%) and cockroach (15%) being the most prevalent. One third of the study population was sensitized to three or less allergens and a similar number displayed sensitization to at least one of the indoor aeroallergens (HDM, cockroach, cat, dog). Sensitization to the mould group of allergens was relatively low (5%) (Table 2).

### 3.1. Serum Fatty Acid Profile

Laboratory analysis for serum fatty acid levels specifically for n-3 and n-6 PUFAs were obtained for 633 subjects (Table 3). Linoleic acid (LA), arachidonic acid (AA) and dihomo-gamma-linolenic acid (DGLA) had the highest mean serum levels among the n-6 PUFAs. Docosahexaenoic acid (DHA) and eicosapentaenoic acid (EPA) had the highest mean serum levels among the n-3 PUFAs.

### 3.2. Asthma Outcomes

While the prevalence of NSBH was 26%, the prevalence of asthma symptoms was much lower (11%) as was current asthma (based on the ECRHS definition), which was 8%. When evaluating asthma outcomes, older age was associated with an increased odds of having asthma symptoms or current asthma. 

Females had a strongly increased odds of having NSBH (OR = 2.71). There was no relationship between current smoking and any of the asthma outcomes (Table 4). In the unadjusted logistic regression models, among the n-6 PUFAs, 18:2 (LA) [OR, 1.14; 95% CI, 1.08–1.21], 20:3 (DGLA) [OR, 1.67; 95% CI, 1.27–2.20] and 20:4 (AA) [OR, 1.18; 95% CI, 1.06–1.31] were positively associated with NSBH (Table 5). The n-6 PUFA 22:2 demonstrated a protective association with respect to the presence of NSBH [OR, 0.29; 95% CI, 0.10–0.87]. However, none of the fatty acids showed any association with atopy. Among the n-3 PUFAs 20:5 (EPA) and 22:5 (DPA) were associated with a decreased risk of having NSBH.

In multivariate logistic regression models adjusting for age, gender and smoking, all the demonstrated associations persisted, confirming that total n-3 fatty acids were associated with a decreased risk while n-6 fatty acids an increased risk of having NSBH (Table 6). However, there were no significant associations between any of the individual n-3 or n-6 PUFAs and asthma risk based on asthma symptoms, although they were all generally in the expected direction of increased risk (n-6 PUFA) or decreased risk (n-3 PUFA).

## 4. Discussion

The prevalence of asthma in this study ranged from 8–11% based on reported asthma symptoms or a standardised definition of current asthma. This is in the same range as estimates from a population-based survey by Ehrlich et al. (2005), which showed the prevalence of recent wheeze to be 13.4%, asthma diagnosis 2.9% and asthma medication usage to be 6.5% among adults aged 20–44 years [37].

The prevalence of NSBH in this population was however much higher at 26%. There was a clear sex difference with respect to the prevalence of NSBH, which was twice as common in women compared to men (30% versus 14%) (Supplementary Table S1). The association between female sex and NSBH has also been reported in previous studies with a threefold higher prevalence in women compared to men in a population-based study in South Africa (OR, 3.6; 95% CI, 1.8–7.0) [38]. Gender differences in asthma epidemiology and severity is well known and is in part related to the difference in immune responses and regulation between the two sexes [39,40,41]. Koper et al. have also suggested a role for sex hormones in their study looking at gender aspects and hormonal influence on bronchial asthma. The study concluded that female sex hormones and their receptors may favour asthma development. Whilst a dose response relationship could not be ascertained, asthma in the perimenstrual period was influenced by dynamic changes in oestrogen levels [42]. In this population, occupational factors such as gender-based job allocations in the seafood-processing industry may also result in women potentially experiencing higher exposures to respiratory irritants and allergens and therefore being more at risk of developing asthma [43]. Of interest too is the high prevalence of smoking among a relatively young predominantly female worker population (62%), which is considerably higher than the population norm of 34% for the South African population at the time of the study [37].

Studies evaluating the relationship between fish intake and serum PUFAs have shown inconsistent results with Welch et al. suggesting that fish and fish oil consumption only accounted for about 25% of the variation in plasma n-3 levels [23]. In contrast other studies. demonstrated that habitual fish intake is reflected in the content of EPA and DHA in serum and in the LDL phospholipid and cholesteryl esters fractions. The blood concentrations of very-long-chain n-3 fatty acids are therefore useful biomarkers for dietary fish intake [22,44]. In a population with high fish consumption and low socioeconomic status, such as this study population, it is likely that marine oils form the main source of n-3 PUFAs in the diet of these communities.

In this study, the n-6 PUFAs dihomo-γ-linolenic acid (DGLA), linoleic acid (LA) and arachidonic acid (AA) were associated with an increase in the risk of NSBH. Woods et al. also demonstrated that DGLA was associated with an increased risk for asthma, current asthma and doctor-diagnosed asthma [24]. The lack of consistency across asthma outcomes in our study compared to that of Woods et al. may in part be due to the different asthma definitions to the former study and the smaller study population. However, the general trend though is consistent with a biologically plausible pro-inflammatory role of certain n-6 PUFAs in the pathogenesis of asthma. Similarly, the inverse association of n-3 PUFAs, with NSBH in particular, suggests that diets rich in EPA and DPA may confer some protection against the development of asthma. This effect is biologically plausible since n-3 PUFAs shunt eicosanoid production away from the arachidonic pathway resulting in a decreased production of bronchoconstrictive leukotrienes [8]. However the conversion from n-3 precursors to long chain PUFAs is not a very active pathway and it is highly recommended to take preformed EPA (plus DHA) from fish or seafood sources.

The relatively high prevalence of atopy in this study is driven in the main by the high prevalence of sensitization to house dust mite generally found among coastal populations. There was no relationship between serum fatty acid levels and atopy in this population. This is similar to the findings by the study of Woods et al. that used a comparable definition of atopy [24]. The review by Sala-Villa et al. concludes that current data do not support the hypothesis that atopy is associated with high n-6 PUFAs levels [45]. Similarly, consistent evidence for the association between low n-3 PUFAs and atopy is also lacking in adults.

The current study allowed for the examination of the relationship between serum fatty acid levels and asthma risk in a coastal working population using various tools including self-reported symptoms, spirometry, blood analysis and non-specific inhalation challenge tests to develop outcome variables for asthma. Whilst the standardised ECHRS definition for current asthma was used, it is recognized that dichotomous definitions of asthma based on symptoms may be less sensitive than using a continuous asthma score. The small sample also resulted in few cases presenting with this outcome of interest which led to a lack of power in the study. The use of specific symptoms furthermore also ignores the fact that asthma is a heterogenous disease with varying phenotypes [27].

The use of spirometry-based diagnoses using NSBH is a strength of this study and represents an attempt to overcome some of the biases related to the use of self-reported symptoms in defining asthma outcomes. Similarly, the measurement and use of serum fatty acid levels as a measure of dietary intake could also be considered a more objective measure than food frequency questionnaires, which are qualitatively imprecise and may perform differently in diverse study settings [46]. 

A recognised limitation of the study was that the categorization of the data collected on seafood and fish consumption did not allow for accurate quantification of the consumption of seafood resulting in difficulty with regard to their interpretation. As a result, this did not allow for the evaluation of the effect of dietary intake on asthma risk. Due to resource constraints, data was not collected on contaminants present in fish. It is known that contaminants such as methylmercury, lead, polychlorinated biphenyls (PCB’s) and perfluorinated chemicals (PFC’s) may negatively influence the potentially beneficial effect of n-3 PUFAs on asthma risk [25]. However, the seafoods most commonly eaten in this population are likely to be very low in mercury and PCBs and therefore contaminants are unlikely to have had a major effect. Whilst there are limited measurements of contaminants and heavy metal content in fish species found in waters of the African coast, the befits of fish and seafood intake generally outweighs the potential risks [47,48]. Furthermore, the lack of information on other potential confounders such as body mass index, recent use of fatty acid supplements, family history of asthma, antioxidant intake and total energy intake may have constrained further analysis of the data [24,46]. This may have introduced a degree of bias with uncertain impact on final estimates generated in this study. However, the study population does represent a fairly homogenous group of workers of relatively low socio-economic status in which fatty acid or antioxidant supplementation is unlikely to occur due to the cost of such supplements. Moreover, Keever et al. in their study of Dutch adults found that supplementation did not confound the relationship between dietary fatty acid intake and FEV1 [24,46]. A further limitation is the cross-sectional nature of the study design, which remains susceptible to reverse causation as temporality cannot be established due to the once-off collection of data. The small sample size may also have rendered the study underpowered and influenced the estimates shown, with some associations occurring due to chance alone. For this reason, the outcome for current asthma based on an ECRHS definition was excluded from the regression analysis (available as Supplementary Material). Since this study was performed in an occupational setting, consideration has to be given to the healthy worker effect and how large an impact this would have had on the prevalence of asthma in this population. Workers with asthma outcomes may have left the industry influencing the prevalence and estimates generated in this study. There was however similarity between the estimates for asthma prevalence obtained in the current study and that found in a national survey, and it was therefore considered unlikely to substantially affect the risk estimates [37].

## 5. Conclusions

This study demonstrated, that n-3 fatty acids (EPA and DPA) are associated with decreased NSBH risk, while certain n-6 fatty acids (LA, DGLA and AA) are associated with an increased risk of NSBH. However, further clarity needs to be obtained on the role of specific PUFAs and how to optimize the intake of long-chain n-3 fatty acids, especially those from fish and seafood sources, to decrease the negative consequences of n-6 fatty acids in influencing asthma outcomes, with a specific focus on important co-factors such as NSBH. A life course approach in such investigations may be more useful since the associations may well differ between children and adults. Further research is needed to evaluate the relationship between serum n-3’s and asthma risk in adults to ascertain whether a protective effect truly exists. Such studies need to include validated questionnaires on intake of foods rich in n-3 PUFA, adequate information on confounders such as body mass index, supplementation nutrients and the possible role of contaminants within fish.

## Figures and Tables

**Table 1:** Subject characteristics and asthma endpoints among fishing village inhabitants along the West Coast of South Africa.

Subject Characteristics	Prevalence (%) Mean/SD (*n* = 642)
**Age (yrs)**	
- Overall	34 (11)
- Female	34 (10)
- Male	35 (12)
**Gender**	
- Female	414 (64%)
- Male	228 (36%)
**Atopic status (%)** (*n* = 625)	234 (37%)
**Mean (SD) % predicted FEV_1_** (*n* = 582)	87 (14)
**Smoking no (%)**	
- Current	328 (51%)
- Ex-smoker	72 (11%)
- Non-smoker	242 (38%)
**Past medical history**	
Previous treatment for tuberculosis	81 (13%)
Previous treatment for chronic bronchitis	61 (9%)
Previous treatment for recurrent chest infections	36 (6%)
Doctor-diagnosed asthma	47 (7%)
**History of allergic disease**	
No. with ocular-nasal symptoms	149 (23%)
No. with hayfever in childhood	30 (5%)
No. on current asthma treatment	29 (5%)
No. on current hayfever treatment	16 (2%)
**Asthma endpoints**	
- Asthma symptoms	72 (11%)
- Current asthma	54 (8%)
- NSBH (*n* = 542)	141 (26%)

**Table 2:** Allergic sensitization profiles of seafood processing workers along the West Coast of the Western Cape.

Sensitization Variables	Prevalence (%) (*n* = 626)
**All aeroallergens**	
Atopy (Positive to at least one aeroallergen)	234 (37%)
**Positive to one aeroallergen**	
House dust mite	155 (25%)
Cockroach	94 (15%)
Rye grass	89 (14%)
Bermuda grass	57 (9%)
Dog	34 (5%)
Mouldmix	22 (4%)
Cat	20 (3%)
*Aspergillus*	14 (2%)
**No. with one to three aeroallergens positive**	211 (32%)
**No. with greater than three aeroallergens positive**	35 (6%)
**At least one indoor allergen** (HDM, cockroach, cat, dog) **positive**	200 (32%)
**At least one pollen allergen** (bermuda grass, rye grass)	98 (16%)
**At least one mould allergen** (mould mix, *Aspergillus*)	33 (5%)

**Table 3:** Serum total phospholipid fatty acid profile (% weight) among fishing village inhabitants along the West Coast of South Africa.

Type of Fatty Acid	Mean PUFA Serum Level * Mean (SD)
**n-3 series PUFAs (%)**	
18:3 ALA	0.07 (0.05)
20:5 EPA	2.10 (1.40)
22.5 DPA	1.11 (0.37)
22.6 DHA	5.65 (1.61)
**n-6 series PUFAs (%)**	
18:2 LA	18.45 (3.78)
18:3 GLA	0.06 (0.07)
20:2	0.39 (0.17)
20:3 DGLA	2.26 (0.74)
20:4 AA	7.89 (1.94)
22:2	0.63 (0.21)
22:4	025 (0.13)
**Mono-unsaturated fatty acids (MUFA)**	
18:1 n-9 (OA)	8.82 (1.66)
**Total**	
n-3	8.93 (2.86)
n-6	29.93 (4.92)

Data are mean (SD) weight % ALA, alpha-linolenic acid; EPA, eicosapentaeoic acid; DPA, docosapentaenoic acid; DHA, docosahexaenoic acid; LA, linolenic acid; GLA, γ-linolenic acid; DHGLA, dihomo-γ-linolenic acid; AA, arachidonic acid; OA, oleic acid.

**Table 4:** Prevalence of asthma endpoints and unadjusted logistic regression models in relation to demographic characteristics among fishing village inhabitants along the West Coast of South Africa.

Prevalence (%)	Asthma Endpoints			
	Asthma Symptoms	Current Asthma (ECRHS)	NSBH ^a^	Atopy
	72/642 (11%)	54/642 (8%)	141/ 542 (26%)	234/626 (37%)
**Age (yrs)**	1.03 (1.00–1.05) *	1.06 (1.03–1.08) ***	1.01 (0.99–1.03)	1.00 (0.99–1.02)
**Female gender**	0.85 (0.52–1.41)	0.86 (0.48–1.52)	2.71 (1.73–4.25) ***	0.95 (0.68–1.32)
**Smoking**				
- Non-smoker	1.00	1.00	1.00	1.00
- Ex-smoker	1.59 (0.76–3.33)	2.83 (1.34–5.99) **	1.63 (0.86–3.09)	0.96 (0.55–1.67)
- Current	0.89 (0.52–1.52)	0.80 (0.42–1.52)	1.32 (0.86–2.02)	1.06 (0.75–1.51)

* *p* ≤ 0.05; ** *p* ≤ 0.01; *** *p* ≤ 0.001. Note: Each OR represents a separate regression model. Associations between serum fatty acid profiles and asthma and atopy. ^a^ NSBH: non-specific bronchial hyperresponsiveness as reflected in a positive methacholine challenge test.

**Table 5:** Unadjusted logistic regression models for asthma endpoints in relation serum phospholipid (% weight) among fishing village inhabitants along the West Coast of South Africa.

Type of Fatty Acid	Asthma Endpoints		
	Asthma Symptoms *n* = 72	NSBH *n *= 141	ATOPY ^a^ *n* = 234
**n-3 series polyunsaturated**			
18:3 ALA	-	0.18 (0.00–15.20)	0.63 (0.17–23.17)
20:5 EPA	0.95 (0.79–1.14)	0.68 (0.56–0.82) ***	0.98 (0.87–1.11)
22:5 DPA	0.87 (0.44–1.73)	0.38 (0.21–0.69) **	1.15 (0.74–1.80)
22:6 DHA	1.06 (0.91–1.23)	1.01 (0.89–1.14)	1.01 (0.91–1.12)
**n-6 series polyunsaturated**			
18:2 LA	0.99 (0.93–1.06)	1.14 (1.08–1.21) ***	1.04 (0.99–1.08)
18:3 GLA	0.12 (0.00–19.41)	1.03 (0.04–25.99)	2.10 (0.19–23.81)
20:2	0.72 (0.13–4.03)	1.38 (0.50–3.82)	1.13 (0.44–2.93)
20:3 DGLA	1.13 (0.82–1.56)	1.67 (1.27–2.20) ***	1.06 (0.86–1.32)
20:4 AA	1.11 (0.98–1.27)	1.18 (1.06–1.31) **	1.04 (0.95–1.13)
22.2	1.28 (0.42–3.84)	0.29 (0.10–0.87) *	0.97 (0.45–2.09)
22:4	1.35 (0.23–8.03)	0.57 (0.12–2.78)	1.52 (0.45–5.12)
**Mono-unsaturated fatty acids (MUFA)**			
18:1 n-9 (OA)	0.97 (0.83–1.14)	0.86 (0.74–0.99) *	1.04 (0.94–1.15)
**Total**			
n-3	1.00 (0.92–1.10)	0.92 (0.85–0.99) *	1.00 (0.95–1.06)
n-6	1.01 (0.96–1.07)	1.14 (1.08–1.21) ***	1.03 (0.99–1.06)

^a^ ATOPY: A positive SPT with a wheal read 15 min after testing that had a diameter (mean of two perpendicular measures) of ≥3 mm more than the negative control to any allergen extract. * *p* ≤ 0.05; ** *p* ≤ 0.01; *** *p* ≤ 0.001. Note: Each OR represents a separate regression model. Associations between serum fatty acid profiles and asthma endpoints.

**Table 6:** Multivariate logistic regression models for asthma endpoints in relation to serum phospholipid (% weight) among fishing village inhabitants along the West Coast of South Africa.

Predictor	Asthma symptoms		NSBH	ATOPY
**n-3 series PUFAs**				
18:3 ALA	-	-	0.16 (0.00–15.72)	0.67 (0.02–25.11)
20:5 EPA	0.95 (0.79–1.14)		0.66 (0.54–0.80) ***	0.98 (0.87–1.11)
22.5 DPA	0.90 (0.45–1.80)		0.37 (0.20–0.69) **	1.16 (0.74–1.81)
22.6 DHA	1.06 (0.91–1.24)		1.01 (0.90–1.15)	1.01 (0.92–1.12)
**n-6 series PUFAs**				
18:2 LA	0.99 (0.93–1.06)		1.14 (1.07–1.22) ***	1.04 (0.99–1.08)
18:3 GLA	0.12 (0.00–19.42)		0.93 (0.04–24.37)	2.12 (0.19–24.08)
20:2	0.75 (0.14–4.11)		1.36 (0.47–3.98)	1.11 (0.43–2.89)
20:3 DGLA	1.14 (0.83–1.57)		1.84 (1.38–2.47) ***	1.06 (0.85–1.32)
20:4 AA	1.12 (0.98–1.27)		1.21 (1.08–1.35) ***	1.04 (0.95–1.13)
22:2	1.41 (0.46–4.32)		0.28 (0.09–0.85) *	0.96 (0.44–2.09)
22:4	1.37 (0.23–8.06)		0.48 (0.10–2.33)	1.48 (0.44–5.02)
**Mono-unsaturated fatty acids (MUFA)**	0.97 (0.83–1.14)	1	0.86 (0.74–0.99) *	(0.94–1.48)
18:1 n-9 (OA)				
**Total**				
n-3	1.00(0.92–1.10)		0.92 (0.85–0.99) *	1.00 (0.95–1.06)
n-6	1.01 (0.96–1.07)		1.14 (1.08–1.21) ***	1.03 (0.99–1.60)

* Adjusted for age, gender and smoking. * *p* ≤ 0.05; ** *p* ≤ 0.01; *** *p* ≤ 0.001. Note: Each OR represents a separate regression model that includes predictor and outcome variables adjusted for age, gender and smoking; the OR is based on 1 unit change in measured serum fatty acid.

## Supplementary Materials

The following are available online at http://www.mdpi.com/1660-4601/16/1/43/s1, Table S1, Adjusted logistic regression models for asthma endpoints stratified by gender and adjusted for age and smoking. Table S2, Unadjusted logistic regression models for asthma endpoints in relation serum phospholipid (% weight) among fishing village inhabitants along the West Coast of South Africa.
