# Supplementary material for: Relationship between Serum Omega-3 Fatty Acid and Asthma Endpoints
Source: Int J Environ Res Public Health. 2018 Dec 25;16(1):43. doi: 10.3390/ijerph16010043 (PMC6338947; doi:10.3390/ijerph16010043)
Supplement: Supplementary file 1 [file ijerph-16-00043-s001.pdf]

Table S1. Adjusted logistic regression models for asthma endpoints stratified by gender and adjusted for age and smoking

Type of fatty acid	NSBH		
	FEMALES	MALES	WHOLE GROUP
18:3 ALA	0.20 (0 – 33.57)	-	0.22 (0.00 – 19.41)
20:5 EPA	0.66 (0.53 – 0.83)***	0.65 (0.42 – 1.02)	
22:5 DPA	0.42 (0.21 – 0.84)*	0.28 (0.07 – 1.10)	0.39 (0.21 - 0.72)**
22:6 DHA	1.03 (0.90 – 1.18)	0.99 (0.74 – 1.32)	1.01 (0.90 – 1.14)
**n-6 series polyunsaturated**			
18:2 LA	1.16 (1.07 – 1.25)***	1.10 (0.98 – 1.23)	
18:3 GLA	0.56 (0.01 – 23.12)	4.16 (0.01 – 2611.66)	1.03 (0.04 – 26.20)
20:2	3.86 (0.68 – 21.73)	0.16 (0 – 7.82)	1.31 (0.47 – 3.66)
20:3 DHGLA	2.04 (1.41 – 2.95)***	1.47 (0.94 – 2.29)	
20:4 AA	1.21 (1.06 – 1.39)**	1.18 (0.96 – 1.44)	1.18 (1.06 – 1.31)**
22.2	0.27 (0.08 – 0.98)*	0.26 (0.02 – 3.17)	0.30 (0.10 – 0.89)*
22:4	0.36 (0.06 – 2.28)	1.16 (0.04 – 31.77)	0.54 (0.11 – 2.64)
**Mono-unsaturated fatty acids (MUFA)**			
18:1n-9 (OA)	0.87 (0.74 – 1.02)	0.83 (0.61 – 1.13)	0.87 (0.76 – 1.00)
**Total**			
n-3	0.93 (0.85 – 1.01)	0.90 (0.75 – 1.07)	0.92 (0.86 – 0.99)*
n-6	1.15 (1.08 – 1.22)***	1.12 (1.01 – 1.24)*	1.14 (1.08 – 1.20)***

*P ≤0.05; ** P ≤0.01; ***P≤ 0.001

Note: Each OR represents a separate regression model

TableS2. Unadjusted logistic regression models for asthma endpoints in relation serum phospholipid (% weight) among fishing village inhabitants along the West Coast of South Africa.

	ASTHMA ENDPOINTS
Type of fatty acid	**Current asthma (ECRHS) N=54**
**n-3 series polyunsaturated**	
18:3 ALA	-
20:5 EPA	0.89 (0.71 – 1.11)
22:5 DPA	0.83 (0.38 – 1.82)
22:6 DHA	1.10 (0.92 – 1.30)
**n-6 series polyunsaturated**	
18:2 LA	1.02 (0.94 – 1.10)
18:3 GLA	1.17 (0.04 – 59.71)
20:2	1.38 (0.34 – 5.60)
20:3 DGLA	0.99 (0.68 – 1.44)
20:4 AA	1.02 (0.88 – 1.17)
22.2	0.93 (0.24 – 3.57)
22:4	0.97 (0.07 – 6.62)
**Mono-unsaturated fatty acids (MUFA)**	
18:1 n-9 (OA)	0.86 (0.70 – 1.05)
**Total**	
n-3	1.00 (0.91 – 1.10)
n-6	1.01 (0.95 – 1.07)

Table S2. Multivariate logistic regression models for asthma endpoints in relation to serum phospholipid (% weight) among fishing village inhabitants along the West Coast of South Africa.

Predictor	Current asthma (ECHRS)
**n-3 series PUFAs**	
18:3 ALA	-
20:5 EPA	0.90 (0.72 – 1.12)
22.5 DPA	0.92 (0.42 – 2.04)
22.6 DHA	1.12(0.94 – 1.33)
**n-6 series PUFAs**	
18:2 LA	1.01(0.94 – 1.09)
18:3 GLA	1.14(0.02 – 68.15)
20:2	1.48(0.37 – 5.93)
20:3 DGLA	1.03(0.71 – 1.5)
20:4 AA	1.03(0.89 – 1.19)
22:2	1.14(0.28 – 4.66)
22:4	0.72(0.07 – 6.98)
**Mono-unsaturated fatty acids (MUFA)**	
18:1 n-9 (OA)	0.85(0.68 – 1.05)
**Total**	
n-3	1.05(0.91 – 1.11)
n-6	1.01(0.95 – 1.07)

* Adjusted for age, gender and smoking

* p ≤ 0.05; ** p ≤ 0.01; ***p ≤ 0.001

Note: Each OR represents a separate regression model that includes predictor and outcome variables adjusted for age, gender and smoking; the OR is based on 1 unit change in measured serum fatty acid
